# Ground state potential energy surfaces around selected atoms from resonant inelastic x-ray scattering

**DOI:** 10.1038/srep20054

**Published:** 2016-01-29

**Authors:** Simon Schreck, Annette Pietzsch, Brian Kennedy, Conny Såthe, Piter S. Miedema, Simone Techert, Vladimir N. Strocov, Thorsten Schmitt, Franz Hennies, Jan-Erik Rubensson, Alexander Föhlisch

**Affiliations:** 1 Institute for Methods and Instrumentation for Synchrotron Radiation Research, Helmholtz-Zentrum Berlin für Materialien und Energie GmbH, Albert-Einstein-Strasse 15, 12489 Berlin, Germany; 2 Institut für Physik und Astronomie, Universität Potsdam, Karl-Liebknecht-Strasse 24/25, 14476 Potsdam, Germany; 3 Max IV Laboratory, Box 118, 22100 Lund, Sweden; 4 FS-Structural Dynamics in (Bio)chemistry, Deutsches Elektronen-Synchrotron, Notkestrasse 85, 22607 Hamburg, Germany; 5 Max Planck Institute for Biophysical Chemistry, Am Faß berg 11, 37077 Göttingen, Germany; 6 Institute for X-ray Physics, Georg-August-Universität Göttingen, Friedrich-Hund-Platz 1, 37077 Göttingen, Germany; 7 Swiss Light Source, Paul Scherrer Institut, 5232 Villigen PSI, Switzerland; 8 Department of Physics and Astronomy, Uppsala University, Box 516, 75120 Uppsala, Sweden

## Abstract

Thermally driven chemistry as well as materials’ functionality are determined by the potential energy surface of a systems electronic ground state. This makes the potential energy surface a central and powerful concept in physics, chemistry and materials science. However, direct experimental access to the potential energy surface locally around atomic centers and to its long-range structure are lacking. Here we demonstrate how sub-natural linewidth resonant inelastic soft x-ray scattering at vibrational resolution is utilized to determine ground state potential energy surfaces locally and detect long-range changes of the potentials that are driven by local modifications. We show how the general concept is applicable not only to small isolated molecules such as O_2_ but also to strongly interacting systems such as the hydrogen bond network in liquid water. The weak perturbation to the potential energy surface through hydrogen bonding is observed as a trend towards softening of the ground state potential around the coordinating atom. The instrumental developments in high resolution resonant inelastic soft x-ray scattering are currently accelerating and will enable broad application of the presented approach. With this multidimensional potential energy surfaces that characterize collective phenomena such as (bio)molecular function or high-temperature superconductivity will become accessible in near future.

The potential energy surface gives a systems potential energy as a function of its different internal degrees of freedom. These degrees of freedom can be nuclear distances and bond angles in molecular systems or lattice constants in crystalline structures. Herewith the electronic ground state potential energy surface provides information on thermally driven chemistry, reaction dynamics and materials functionality. The interplay of local modifications at active centers, impurities and doping with the long range structure of the potential energy surface drives collective phenomena such as (bio)molecular function and high temperature superconductivity^1^,^2^,^3^,^4^,^5^,^6^. To determine experimentally the electronic ground state potential energy surface around selected atomic sites requires the elemental and chemical state selectivity of resonant soft x-ray spectroscopy.

Resonant inelastic x-ray scattering (RIXS)^7^,^8^,^9^ probes the energy and dispersion of electronic, magnetic, orbital and spin excitations of matter and finds wide application in materials science and physical chemistry^3^,^4^,^10^,^11^,^12^. Advances in instrumentation^13^,^14^,^15^ give now increasingly access to the relevant meV energy scales of these fundamental low-energy excitations^3^,^16^,^17^,^18^. RIXS as a coherent Raman scattering process preserves both energy and bandwidth of the scattered radiation. Therefore resonant scattering with the electronic ground state as initial and final state experiences no broadening from the femtosecond lifetime of the core-excited intermediate state and no final state lifetime broadening since the final state is the electronic ground state. Thus, using an incident bandwidth narrower than the intermediate state lifetime broadening (sub-natural linewidth), RIXS is uniquely suited to pick up low energy vibrational progressions with the elemental and chemical selectivity of the atomic soft x-ray scattering resonance^16^,^17^,^19^,^20^,^21^,^22^,^23^. This principle is illustrated in Fig. 1 where the schematic scattering process is shown together with experimental examples of chemically bound oxygen atoms in non-interacting, weakly interacting and strongly interacting molecular as well as solid state environments. Independent of interaction strength, vibrational resolution with a total linewidth far below the oxygen K-edge natural linewidth of 150 meV^19^ is achieved.

Figure 1(a) illustrates how resonant core level excitation projects the ground state nuclear wave function onto the core-excited state potential energy surface where it propagates during the femtosecond core hole lifetime. The propagated wave packet reaches Franck-Condon overlap with a multitude of vibrational excited states in the electronic ground state giving rise to a vibrational progression in the RIXS spectrum. Here the vibrational progression reflects the electronic ground state potential energy surface along a set of coordinates along which the nuclear wave packet has propagated in the core-excited state. Experimental results are summarized in Fig. 1(b). Non-interacting diatomic O_2_ in the gas phase has only one vibrational mode, namely the O = O stretch^19^. For weakly interacting liquid acetone the excitation into the C = O anti-bonding π^*^ orbital drives predominantly the C = O stretch mode out of all present normal modes of acetone^21^. In the strongly hydrogen bonded network of liquid water as well as for the layered mineral kaolinite resonant excitation into a state with O–H anti-bonding character predominantly drives the local O–H stretch modes which are observed as quasi-collective modes due to internal coupling. Other normal modes in these systems are seen as sub-structure in the individual vibrational peaks. Hence, the resonant excitation scheme allows to individually address and clearly assign specific internal degrees of freedom also in complex molecules and interacting systems. These findings illustrate firstly that vibrational RIXS onto the ground state potential energy surface is independent of the core-excited state lifetime broadening^7^,^9^. Secondly vibrational RIXS on the ground state potential energy surface does not suffer from dipolar environmental broadening, since the initial and final electronic state are identical^21^. This means that the vibrational structure of the electronic ground state can be measured by RIXS with an accuracy that is determined exclusively by the bandwidth of the scattered radiation.

Let us now focus onto the question how well a weak local perturbation to the potential energy surface can be detected and quantified. To this end, we compare the potential energy surface around the oxygen atoms of weakly interacting acetone molecules in the liquid phase to the case where a slight perturbation due to C = O · · · H hydrogen bond formation between the oxygen atom of acetone and the hydrogen atom of chloroform is introduced in the negative azeotrope acetone-chloroform complex^24^.

In Fig. 2(a) we show the vibrational progression in the electronic ground state potential of acetone and the hydrogen bonded acetone-chloroform complex for resonant oxygen 1*s* to LUMO 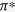 excitation (see Methods and Materials section for the x-ray absorption spectra). Due to the C = O anti-bonding character of the LUMO 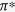 orbital the vibrational progression is dominated for both samples by the C = O stretch mode 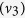 with a fundamental frequency of ~210 meV. Through the energy spacing of the overtones, the vibrational progression contains the shape of the ground state potential energy surface along the C = O coordinate. To extract the shape in this molecular system, we choose the Morse potential and set up a simple model with vertical Franck-Condon transitions (see Methods and Materials section for details). The global parameterization of all vibrational peak positions in the progression by only two parameters provides a robust fit model and enables us to extract the small differences in the peak positions at higher overtones that are hardly recognizable in the raw data, Fig. 2(b). The two parameters in the Morse potential model characterize at the same time the complete potential energy curve of the electronic ground state along the C = O internuclear distance, Fig. 2(d). Extrapolation of the potentials to internuclear distances and potential energies beyond the point where the vibrational progression is observed in the spectra is directly possible with an accuracy that is solely determined by the signal-to-noise ratio and spectral resolution of the experimental data. Enhanced spectral resolution will enable the observation of additional vibrational modes, which will make further coordinates of the potential energy surface accessible.

In a first step we modeled the vibrational progression of acetone and the acetone-chloroform complex while optimizing both parameters in the Morse potential within a nonlinear least-squares fitting procedure. In this case a slightly larger fundamental C = O vibrational energy (resulting in a slightly narrower potential) but at the same time a significantly larger asymmetry were obtained for the potential of the acetone-chloroform complex as compared to acetone. The resulting differences in peak positions of the higher overtones are illustrated by the filled markers in Fig. 2(b) and the constructed Morse potentials are given by the thick colored lines in Fig. 2(d). To assure that the comparably large difference in asymmetry is not an analysis artifact that overcompensates the slight narrowing of the potential from the larger fundamental vibrational energy, we fixed in a second step the fundamental vibrational energy in the fit for the acetone-chloroform complex to the value obtained for acetone. In this case the effect on the asymmetry is reduced, making the potential of the acetone-chloroform complex more similar to the one of acetone [open symbols in Fig. 2(b) and thin green line in Fig. 2(d)]. However, the general trend of a larger asymmetry for the acetone-chloroform complex is preserved. The variation between the fit with and without fixed vibrational energy gives an estimate for the accuracy of the used model. Note that the goodness-of-fit values resulting from the two models are identical (see Table 1 in the Methods and Materials section).

In light of the comparable large uncertainties in the derived potentials, we do not aim at a detailed quantitative comparison between the potentials of acetone and the hydrogen bonded acetone-chloroform complex. However, we observe, independently of the used fit model, a tendency towards softening of the C = O potential for the acetone-chloroform complex [Fig. 2(d)]. This is the expected trend upon C = O · · · H hydrogen bond formation^25^ and in line with direct infrared measurements of the fundamental C = O vibrational energy where a small shift to lower wavenumbers was observed for the acetone-chloroform complex^26^. Furthermore density functional theory calculations of the C = O potential energy curves confirm this trend and the small order of magnitude of the change induced by the hydrogen bond (see Supporting Information). The parameter 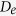 of the Morse potential (see Methods and Materials section) is directly related to the bond dissociation energy. From the fit to the acetone spectrum in Fig. 2 we determine a C = O bond dissociation energy in acetone of 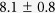 eV, which compares well with literature value of 7.7 eV^27^ and shows the quality and reliability of the comparably simple Morse potential approach. However, to address structures and materials with an increasing degree of complexity and functionality and to determine multidimensional potential energy surfaces we point out the need for more advanced potential models and in particular of ab initio derived potential energy surfaces to compare with experimental results. In addition to accessing the ground state potential energy surface with the here presented approach, certain low lying electronic excited final states show clearly resolved vibrational progressions^19^, which will provide potential energy surfaces of photochemical relevant low energy electronic excited states. Finally, potential energy surfaces of ionic species are accessible by high-resolution electron spectroscopy^28^,^29^.

In conclusion, we demonstrated how sub-natural line width vibrational resolved RIXS gives direct experimental access to the ground state potential energy surface around selected atomic sites and moieties, not accessible with other techniques. Our approach to the local ground state potential energy surface at the relevant sites and moieties within a large molecular aggregate or in condensed matter resembles finding a needle in a haystack. The performance of this approach is conceptually open to even smaller energy scales and will benefit strongly from upcoming high-brilliance synchrotrons and free-electron lasers in combination with upcoming high resolution RIXS instruments. Therefore, we foresee wide applicability of this technique to all thermal, collective and impurity driven chemistry and materials issues in the near future. Multidimensional potential energy surfaces that characterize collective phenomena such as (bio)molecular function or high temperature superconductivity should become accessible with the ongoing instrumental developments in combination with ab initio derived potential energy surfaces.

## Methods and Materials

RIXS experiments have been performed at the ADRESS - X03MA beamline^30^ at the Swiss Light Source, Paul Scherrer Institut, Villigen, Switzerland, using the SAXES spectrometer^14^ with a combined spectral resolution of 50–60 meV in our measurements. Gaseous and liquid samples were prepared in a flow-cell with a 100 nm thick diamond-like window coated with 10 nm gold on the inside. The sample in the interaction region was renewed at a rate of 15 Hz. Linearly polarized x-rays were used with the polarization vector perpendicular to the scattering plane defined by the incident x-ray beam and the direction of detection.

Absolute energy calibration of the incident photon energy was done using the position of the most intense 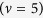 vibrational substate of the carbon monoxide x-ray absorption resonance (measured in total ion yield) at 534.15 eV given by Prince *et al.*^31^. The emission energy scale of the RIXS spectra was calibrated using the vibrational progression of the O_2_ ground state and the vibrational energies given by Cheung *et al.*^32^.

**Figure 2:**
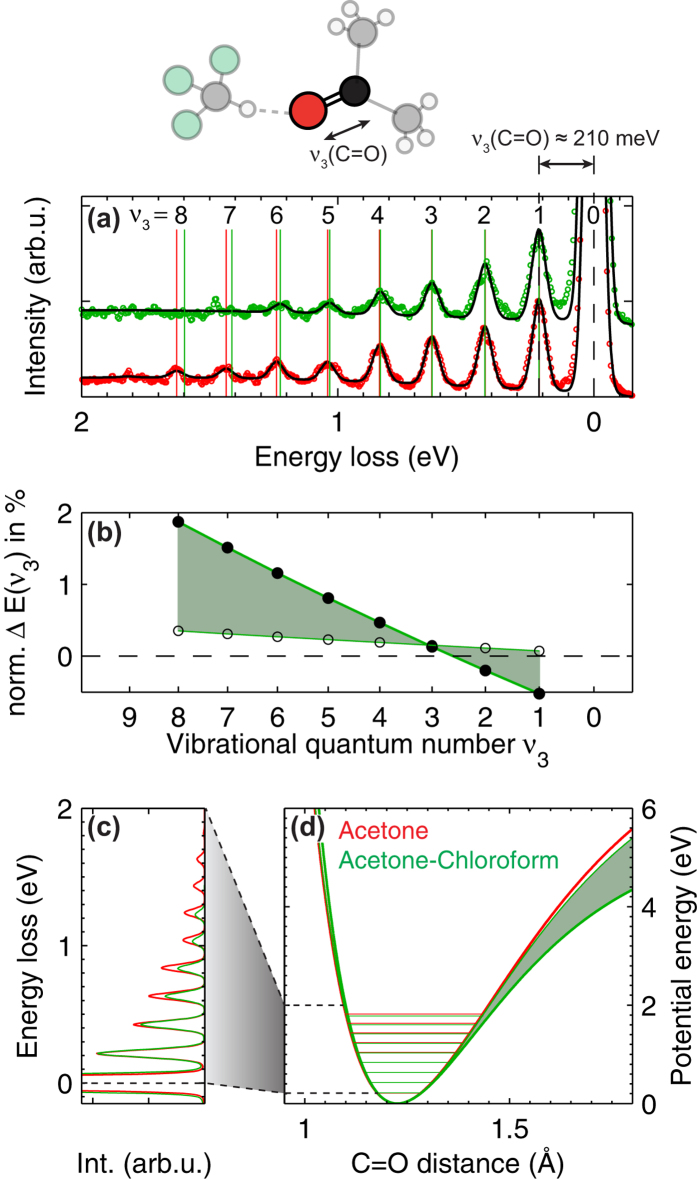
Reconstruction of ground state potential energy surfaces from the experimental vibrational progressions. Softening of the potential around the oxygen atom of acetone due to hydrogen bond formation is observed. (**a**) Ground state vibrational progression in acetone and the acetone-chloroform complex. Experimental data (colored markers) and fitted curves (solid black lines) resulting from the fit when optimizing both Morse parameters are shown. (**b**) Normalized differences between peak positions in acetone and the acetone-chloroform complex. Filled symbols = Optimizing both Morse parameters. Open symbols = Fundamental vibrational energy fixed. See main text and methods section for details. (**c**) Direct comparison of the fitted curves from (**a**). (**d**) C = O potential energy curves resulting from the fit to the experimental data. For the acetone-chloroform complex the thick line results from the fit where both Morse parameters are optimized and the thin line to the fit where the fundamental vibrational energy is fixed.

**Figure 3:**
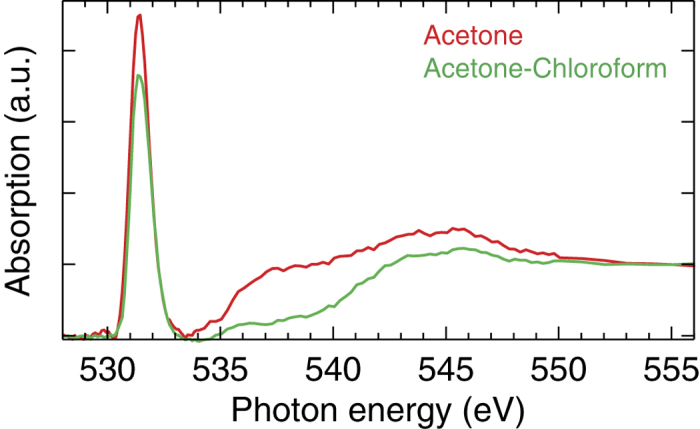
Oxygen K-edge x-ray absorption spectra of acetone and the hydrogen bonded acetone-chloroform complex. Both samples feature the dominating 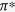 resonance at 531.5 eV that has C = O anti-bonding character.

In Fig. 3 we show the oxygen K-edge x-ray absorption spectra of acetone and the hydrogen bonded acetone-chloroform complex. Both spectra are dominated by the prominent LUMO 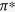 resonance at 531.5 eV, which was the scattering resonance for extracting the vibrational progressions in Fig. 2. (In the acetone-chloroform complex, the LUMO is of *π* character, but strictly speaking not a 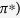. The established hydrogen bond reduces the *π* character of the C = O bond in the acetone-chloroform complex, which is directly reflected in reduced intensity of the 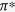 peak in Fig. 3.

The x-ray absorption spectra of liquid acetone and the liquid acetone-chloroform azeotrope were measured at beamline UE52-SGM at the synchrotron radiation source BESSY II of the Helmholtz-Zentrum Berlin, Germany. We used a specialized sample holder^33^ to measure XA spectra in transmission mode from sub μm thin liquid films. Spectra were measured for different sample thicknesses to ensure the absence of saturation effects.

Liquid acetone (99.8%, anhydrous) was purchased from Sigma-Aldrich and used as received, but special care was taken to minimize contact to air. The acetone-chloroform azeotrope mixture was distilled from acetone 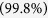 and chloroform 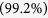, both purchased from Merck Chemicals, at the azeotropic boiling point of 64.7 °C. During the distillation the refractive index of the azeotrope mixture was monitored to ensure successful formation of hydrogen bonded acetone-chloroform complexes. Deionized and properly degassed liquid water was used. The natural kaolinite (Fluka Analytical, Al_2_O_7_Si_2_) was purchased from Sigma-Aldrich and pressed into a pellet.

The fit model used in Fig. 2 was a sum of pseudo-Voigt profile peaks using a parameterization which allows an independent lorentzian and gaussian width^34^. The gaussian width (instrumental broadening) was determined by fitting the elastic line (zero energy loss) and then kept fixed for the fit of the vibrations. The lorentzian width was a free fit parameter, but same for each vibrational peak within the progression. The gaussian and lorentzian width were determined in the fit to (60 ± 5) meV and (5 ± 1) meV for both spectra. To model the background of the vibrations that results from different overlapping low energy modes forming a quasicontinuum^21^ a second order polynomial was added.

The peak positions were parameterized using the energy levels of the Morse potential. I.e. the position of the *n*-th peak 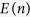 in the vibrational progression (with 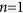 for the first vibrational loss) was given by:


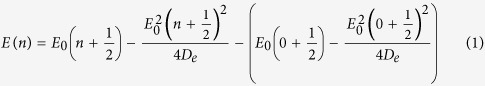


With 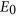 and 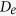 being free fit parameters and characterizing the Morse potential. The values obtained from the fit for acetone and for the acetone-chloroform complex with and without fixed fundamental vibrational energy 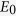 as well as the adjusted R-square values of the nonlinear least-squares fitting procedure are given in Table 1.

The amplitude of each vibrational peak was a free fit parameter. Note, though, that the amplitudes of the vibrational peaks as well as the intensity of the background modeled by the 2nd order polynomial are independent of the shape of the ground state potential energy surface. The shape of the ground state potential energy surface is exclusively determined by the positions of the vibrational peaks.

To reconstruct the Morse potentials displayed in Fig. 2(d) the following parameterization was used


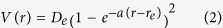


The parameter *a* defines the asymmetry of the Morse potential and is related to the fit parameters *E*_0_ and 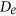 via 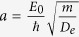. *m* is the reduced mass of the diatomic molecule. The reduced mass of C = O was used. 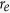 is the equilibrium bond distance, which was set to the literature value of acetone 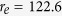 pm^35^.

## Additional Information

**How to cite this article**: Schreck, S. *et al.* Ground state potential energy surfaces around selected atoms from resonant inelastic x-ray scattering. *Sci. Rep.* **6**, 20054; doi: 10.1038/srep20054 (2016).

## Supplementary Material

Supplementary Information

## Figures and Tables

**Figure 1:**
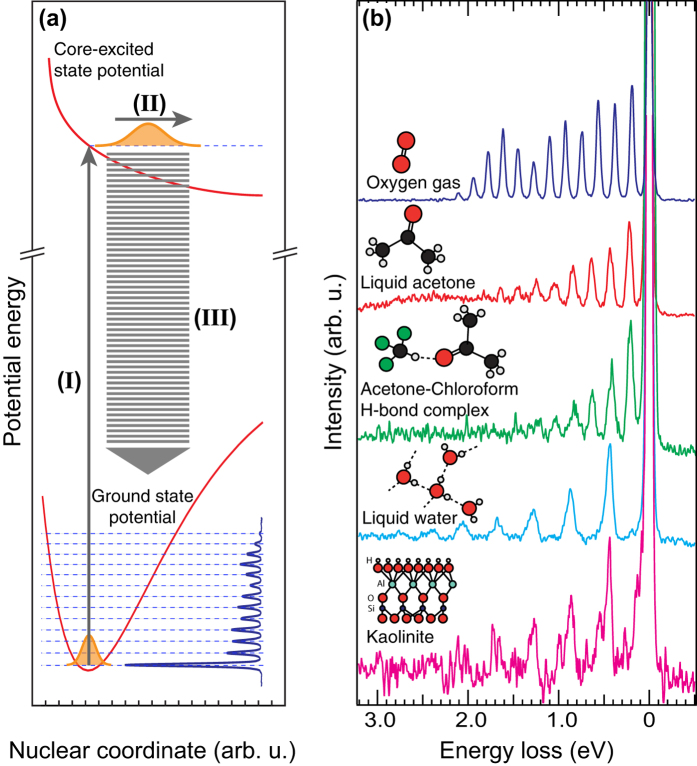
Sub-natural line width resonant inelastic x-ray scattering and the electronic ground state potential energy surface. (**a**) Schematic illustration of the scattering process into purely vibrational excited final states in the electronic ground state. See also main text. (**b**) Experimental findings as a function of increasing degree of interaction. The O_2_ data from Hennies *et al.*^19^, measured at resonant 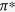 excitation (530.5 eV in O_2_). The acetone and acetone-chloroform spectra were also measured at resonant 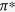 excitation (531.5 eV in acetone and acetone-chloroform). The liquid water spectrum from Pietzsch *et al.*^23^ was measured at resonant pre-edge excitation at 534.9 eV excitation energy. The kaolinite spectrum was measured at 534.7 eV excitation energy.

**Table 1:** Parameter values and uncertainties obtained from the fit to the vibrational progressions in Fig. 2 that determine the Morse potential.

	*D*_*e*_	*E*_0_	adj. *R*^2^
Acetone	8.1 ± 0.8 eV	216.6 ± 0.8 meV	0.9989
Acetone-Chloroform	5.6 ± 1.8 eV	219.1 ± 2.9 meV	0.9991
Acetone-Chloroform (fixed *E*_0_)	7.7 ± 1.3 eV	Fixed at 216.6 meV	0.9991
