# Erratum: Ground state potential energy surfaces around selected atoms from resonant inelastic x-ray scattering

**DOI:** 10.1038/srep46386

**Published:** 2017-06-30

**Authors:** Simon Schreck, Annette Pietzsch, Brian Kennedy, Conny Såthe, Piter S. Miedema, Simone Techert, Vladimir N. Strocov, Thorsten Schmitt, Franz Hennies, Jan-Erik Rubensson, Alexander Föhlisch

Scientific Reports 6: Article number: 20054; 10.1038/srep20054 published online: 01 29 2016; updated: 06 30 2017.

The original HTML version of this Article listed an incorrect volume number. This has now been corrected in the HTML version; the PDF version was correct at the time of publication.

