# Supplementary material for: Ground state potential energy surfaces around selected atoms from resonant inelastic x-ray scattering
Source: Sci Rep. 2016 Jan 29;7:20054. doi: 10.1038/srep20054 (PMC4731820; doi:10.1038/srep20054)
Supplement: Supplementary Information [file srep20054-s1.pdf]

Supporting Information:
Ground state potential energy surfaces around selected atoms from resonant inelastic x-ray scattering

Simon Schreck[1,2], Annette Pietzsch[1], Brian Kennedy[1], Conny Såthe[3], Piter S. Miedema[1], Simone Techert[4, 5, 6], Vladimir N. Strocov[7], Thorsten Schmitt[7], Franz Hennies[3], Jan-Erik Rubensson[8], and Alexander Föhlisch[1, 2]

[1]*Institute for Methods and Instrumentation for Synchrotron Radiation Research, Helmholtz-Zentrum Berlin für Materialien und Energie GmbH, Albert-Einstein-Strasse 15, 12489 Berlin, Germany*
[2]*Institut für Physik und Astronomie, Universität Potsdam, Karl-Liebknecht-Strasse 24/25, 14476 Potsdam, Germany*
[3]*Max IV Laboratory, Box 118, 22100 Lund, Sweden*
[4]*FS-Structural Dynamics in (Bio)chemistry, Deutsches Elektronen-Synchrotron, Notkestrasse 85, 22607 Hamburg, Germany*
[5]*Max Planck Institute for Biophysical Chemistry, Am Faßberg 11, 37077 Göttingen, Germany*
[6]*Institute for X-ray Physics, Georg-August-Universität Göttingen, Friedrich-Hund-Platz 1, 37077 Göttingen, Germany*
[7]*Swiss Light Source, Paul Scherrer Institut, 5232 Villingen PSI, Switzerland.*
[8]*Department of Physics and Astronomy, Uppsala University, Box 516, 75120 Uppsala, Sweden*

S1 Calculated C=O potential energy curves of acetone and the acetone-chloroform complex

In Figure S1 we present calculated potential energy curves along the C=O bond distance of acetone and the acetone-chloroform complex. A small but clear effect of the hydrogen bond in the acetone-chloroform complex can be observed. This effect is in line with the trend observed in the potentials extracted from the experimental data in the main paper.

The curves in Figure S1 were calculated using the ORCA software package [1]. For this, in a first step, the acetone molecule and acetone-chloroform complex were geometry optimized using the BP86 functional and def2-TZVP basis sets for all atoms. For the optimized structures, the potential energy surface was scanned along the C=O bond distance coordinate using the standard ORCA routine with the same functional and basis sets as for the geometry optimization.

References

[1] F. Neese. The ORCA program system. *Wiley Interdisciplinary Reviews: Computational Molecular Science*, 2(1):73–78, Jan. 2012.

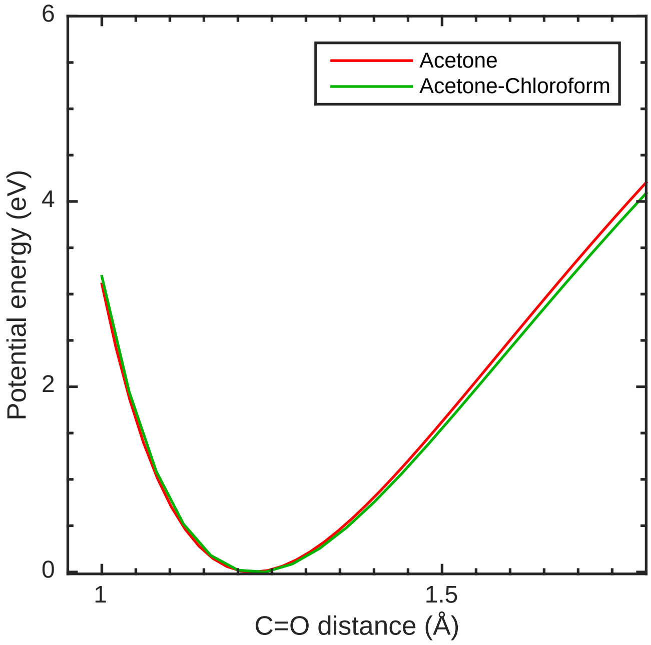

Figure S1: Calculated C=O potential energy curves for acetone and the hydrogen bonded acetone-chloroform comlex.
